# Diphtheria

**Published:** 1875-04

**Authors:** 


### DIPHTHERIA.

The following report of the Sanitary Committee of the Board of Health of the City of New York, is approved and published by the Board. We publish it for the benefit of our readers, and recommend it to their careful consideration. Diphtheria is one of the most dangerous and fatal diseases with which we have to contend, and any means for its suppression should be ardently practised by an intelligent public.

"*Mode of Attack.*—Diphtheria is caused by the innoculation of the air passages with the diphtheritic poison, which from this poisons the whole system; the local inflammation is attended by the formation of membrane, (exudation), the fever and general symptoms are the result of this local infection.

"*How it Spreads.*—Diphtheria is therefore a contagious disease, (not perhaps as marked as scarlet fever), induced by contact with persons and objects infected. It may be diffused by the exhalations of the sick, by the air surrounding them, or directly by exudation, communicated in the act of kissing, coughing, spitting, sneezing, or by the infected articles used, as towels, napkins, handkerchiefs, etc. The poison clings with great tenacity to certain places, rooms, and houses, where it may occasion cases after the lapse of months.

*Symptoms.*—In ordinary attacks the poison begins to act the moment it lodges upon the tissues, but, like a vaccination, causes but slight sensible effects in from two to five days; then there is marked prostration, dryness of the throat, and pricking pain in swallowing; the throat becomes red, and patches of white exudation appear, and the glands of the neck swell. In mild cases these symptoms subside on the third or fourth day from their appearances; if more severe, these symptoms may be prolonged; if unfavorable the fever increases, the local inflammation spreads, and exhaustion rapidly follows.

"*Predisposing Conditions—The Persons.*—Diphtheria attacks by preference children between the ages of one and ten years, (the greatest mortality being in the second, third and fourth year); children of feeble constitution, and those weakened from previous sickness; and those suffering from catarrh, croup, and other forms of throat affections.

"*Social Relations.*—All classes are liable to diphtheria where it is prevailing, but those suffer most who live on low, wet ground; in houses with imperfect drains or surrounded by offensive matter, as privies, decaying vegetable or animal matter; in damp rooms, as cellars; in overcrowded and unventilated apartments.

"*Seasons.*—Diphtheria is not affected by either heat or cold, drought or rain.

"*Precautions.* (*a.*) *The Dwelling or Apartment.*—Cleanliness in and around the dwellings, and pure air in living and sleeping rooms, are of the utmost importance where any contageous disease is prevailing, as cleanliness tends to prevent and mitigate it. Every kind and source of filth around and in the house should be thoroughly removed; cellars and foul areas should be cleansed and disinfected; drains should be in perfect order; dirty walls and ceilings should be lime washed, and every occupied room thoroughly ventilated. Apartments which have been occupied by persons sick with diphtheria should be cleansed with disinfectants; ceilings lime washed, and wood-work painted; the carpets, bed clothing, upholstered furniture, etc., exposed many days to fresh air and sunlight, (all articles which may be boiled or subjected to high degrees of heat should be thus disinfected); such rooms should be exposed to currents of fresh air for at least one week before re-occupation.

"(*b.*) *When Diphtheria is Prevailing.*—No child should be allowed to kiss strange children nor those suffering from sore throat, (the disgusting practice of compelling children to kiss every visitor is a well contrived method of propagating other graver diseases than diphtheria); nor should it sleep with nor be confined to rooms occupied by, or use articles, as toys, taken in the mouth, handkerchiefs, etc., belonging to children having sore throat, croup or catarrh. If the weather is cold the child should be warmly clad with flannels.

"(*c.*) *When Diphtheria is in the House or Family.*—The well children should be scrupulously kept apart from the sick, in dry, well aired rooms, and every possible source of infection through the air, by personal contact with the sick, and by articles used about them or in their rooms, should be rigidly guarded. Every attack of sore throat, cough and catarrh should be at once attended to; the feeble should have invigorating food and treatment.

"(*d.*) *Sick Children.*—The sick should be rigidly isolated in well aired, (the air should be entirely changed at least hourly), sun-lighted rooms, the outflow of air being, as far as possible, through the external windows by depressing the upper and elevating the lower sash, or a chimney heated by a fire in an open fire-place. All discharges from the mouth and nose should be received into vessels containing disinfectants, as solutions of carbolic acid, or sulphate of zinc; or upon cloths which are immediately burned; or if not burned, thoroughly boiled, or placed under a disinfecting fluid."

